# Elucidating the impact of obesity on hormonal and metabolic perturbations in polycystic ovary syndrome phenotypes in Indian women

**DOI:** 10.1371/journal.pone.0246862

**Published:** 2021-02-26

**Authors:** Roshan Dadachanji, Anushree Patil, Beena Joshi, Srabani Mukherjee

**Affiliations:** 1 Department of Molecular Endocrinology, ICMR-National Institute for Research in Reproductive Health, Parel, Mumbai, India; 2 Department of Clinical Research, ICMR-National Institute for Research in Reproductive Health, Parel, Mumbai, India; 3 Department of Operational Research, ICMR-National Institute for Research in Reproductive Health, Parel, Mumbai, India; Zhejiang University School of Medicine Women’s Hospital, CHINA

## Abstract

Polycystic ovary syndrome is a complex endocrinopathy with heterogeneous presentation and multifactorial etiology. We have undertaken this case-control study to compare metabolic and endocrine characteristics in different phenotypic subgroups of women with PCOS and the impact of obesity on them. Women with PCOS (n = 489) were classified into 4 phenotypes according to Rotterdam criteria. Comparisons of clinical, biochemical and hormonal parameters were performed across all phenotypic groups of PCOS and with controls (n = 270) by Welch’s ANOVA with subsequent Games-Howell post-hoc test. We found maximum prevalence of normoandrogenic phenotype D, which is milder form of PCOS in terms of insulin resistance, gonadotropin levels and dyslipidemia, followed by phenotype A, in our total study population. After classification of the study group into lean and obese groups, only few insulin and lipid-related traits showed marked differences between phenotypes. Further, we noted that obese women showed adverse metabolic but not androgenic traits compared to lean counterparts in the same phenotype. Metabolic syndrome frequency is increased in hyperandrogenic phenotypes with HDL-C and waist circumference being most predominant contributing factors in total, lean and obese groups. We demonstrate that in our study population there is greater occurrence of phenotype D of PCOS. Our study highlights the importance of clinicians concurrently employing Rotterdam criteria along with obesity status for ascertaining accurate PCOS status and formulating suitable therapeutic intervention.

## Introduction

Polycystic ovary syndrome (PCOS) is a multifactorial complex endocrinopathy characterized by irregular menses, anovulatory infertility, hirsutism, acne, altered LH:FSH ratios, augmented androgen production and polycystic ovaries on ultrasound examination [1]. Affected women frequently present with aggravated insulin resistance and dyslipidemia, leaving them susceptible to development of metabolic sequelae such as glucose intolerance, type 2 diabetes (T2D), metabolic syndrome and cardiovascular disease [1,2]. Apart from hormonal and metabolic perturbations, genetic factors are substantially involved in modifying PCOS susceptibility which have been elucidated by twin, candidate gene and genome-wide association studies [2].

Obesity is often observed in women with PCOS with predominant abdominal obesity. Obesity exacerbates both the abovementioned metabolic sequelae and reproductive aberrations including menstrual disturbances, adverse pregnancy outcomes and poor response to infertility treatment [3]. Visceral adiposity index and lipid accumulation product are mathematically calculated clinical markers for assessing accumulation of excessive abdominal fat and fat distribution respectively, which have been proposed to serve as robust indicators of onset of insulin resistance, metabolic syndrome and cardiovascular disease [4,5]. The correlation of obesity and visceral adiposity with alterations in adipose tissue gene expression profiles, lipid metabolism and steroid hormone profiles [6–8] further accentuates its impact on PCOS pathogenesis.

Several expert committees have proposed diagnostic criteria for PCOS after much deliberation. According to the National Institute of Health 1990 guidelines, PCOS diagnosis required the presence of chronic anovulation associated with clinical and/or biochemical signs of androgen excess [9]. The 2003 consensus workshop by European Society of Human Reproduction and Embryology (ESHRE)/American Society for Reproductive Medicine (ASRM) in Rotterdam widened the NIH criteria to include polycystic ovary morphology [10]. As PCOS women show heterogeneous presentation, sub-categorizing them by combinations of these criteria identified three hyperandrogenic and one-non-hyperandrogenic phenotypes with varying degrees of metabolic and endocrine severity [9]. Hyperandrogenic phenotypes are generally connected with greater degree of insulin resistance and unfavorable metabolic aberrations. The Androgen Excess Society upheld that PCOS was mainly attributed to androgen excess, and their 2006 criteria entailed presence of clinical and/or biochemical hyperandrogenism accompanied by either ovarian dysfunction and/or PCO [9].

Recently, it has been demonstrated that PCOS prevalence differs in Chinese, white, and Black women, highlighting the necessity of formulating ethnicity specific guidelines [11,12]. It was further reported that South Asian, African American, Asian Indians and Hispanic women with PCOS commonly present with more adverse metabolic perturbations, while Middle-Eastern and Mediterranean women are generally more hirsute [11]. India itself is an ethnically diverse country comprising many subpopulations based on geographical location attributed to genetic and environmental dissimilarities which in turn may influence presence of hyperandrogenism and insulin resistance and the prevalence of PCOS sub-phenotypes. Interestingly, a multi-country study has observed that Indian women had maximum predisposition to metabolic syndrome followed by US and Norwegian women with PCOS [13]. A noteworthy study on Indian women from two regions of Northern India further highlights influence of geographic variation on phenotypic manifestation by showing distinct phenotypes in two cities of North India viz., lean hyperandrogenic women from Srinagar and obese hyperinsulinemic women from Delhi [14].

The present study has sought to investigate the metabolic and hormonal patterns in different phenotypic subgroups of PCOS in women from Western India to elucidate the pathogenesis of this heterogeneous disorder. As these traits are affected by obesity, we have further compared these traits after subgrouping our study population into BMI-matched groups for different phenotypes, which remains relatively unexplored.

## Materials and methods

### Study participants

Four hundred and eighty-nine women were diagnosed with PCOS according to Rotterdam consensus diagnosis criteria satisfying at least two of the following three features; (i) oligo or anovulation (OA) (ii) clinical and/or biochemical hyperandrogenism (HA), and (iii) polycystic ovaries morphology (PCO). Women presenting with hyperprolactinemia, thyroid dysfunction, Cushing syndrome, congenital adrenal hyperplasia and androgen producing neoplasm were excluded using appropriate clinical and laboratory tests. Additionally, we recruited 270 regularly menstruating women from the same local community showing no clinical or biochemical signs of hyperandrogenism and having normal ovaries on ultrasound. The study was approved by the human ethics committee at National Institute for Research in Reproductive Health. Participants were recruited from clinic at National Institute for Research in Reproductive Health, Mumbai, India, and written consent was obtained from all participants prior to enrollment in the study. No participant had taken any medicines for the last three months that could alter their carbohydrate and lipid metabolism or endocrine parameters.

### Classification of study participants

Women with PCOS were assigned into the four phenotypes on basis of Rotterdam criteria, into phenotype A (HA+OA+PCO), phenotype B (HA+OA), phenotype C (HA+PCO) and phenotype D (OA+PCO) [9]. HA was defined in terms of biochemical hyperandrogenism, as women having free androgen index (FAI) greater than 4.13, which is 95^th^ percentile value of free androgen index (FAI) estimated in control women. Clinical signs of hyperandrogenism were only marked by observation and self-reporting by the patient who frequently made use of hair removal and acne reducing treatments and answered only as yes or no. However, these cannot be relied upon and could not be accurately assessed by trained clinician as women would use common hair removal methods on a regular basis. Acanthosis nigricans, a surrogate marker for insulin resistance was also evaluated as either presence or absence of black velvety patches in body folds and creases. OA was defined as cycle lengths >35 days for oligomenorrheic women and >3 months for secondary amenorrheic women. Transabdominal ultrasound was carried out in women who had not commenced sexual activity, and transvaginal ultrasonography was performed for sexually active women using high-resolution Philips HD 5 with a 3.5 Hz convex-array Probe. PCO was defined as the presence of ≥12 follicles, measuring 2–9 mm, and/or an ovarian volume >10 cm^3^. Given that obesity impacts key cardiometabolic and hormonal traits and that many participants are overweight/obese, we also categorized study participants according to body mass index as per guidelines for Indian women, as lean (BMI <23kg/m^2^) and overweight/obese (BMI ≥23kg/m^2^) henceforth referred as obese group [15].

### Estimation of clinical, biochemical and hormonal parameters

The anthropometric data–height, weight, BMI, waist to hip circumference ratio (WHR), and blood pressure were obtained. Except for secondary amenorrheic women with PCOS, blood was collected from all participants in the early follicular phase (days 3–7 of their menstrual cycle) following an overnight fast. Fasting serum was stored at −80 °C until assayed for hormonal and biochemical parameters.

Each subject underwent an oral glucose tolerance test (OGTT) which estimates the risk of prediabetes and T2D. Glucose levels were measured at fasting and 2 h after 75g of glucose load from plasma by glucose oxidase method. Complete lipid profiling of subjects measuring total cholesterol, triglycerides (TG), high-density lipoprotein cholesterol (HDL-C), apolipoprotein A-1 (Apo A-1) and apolipoprotein B (Apo B) was carried out on an automated biochemistry analyzer (Erba 200, Mannheim, Germany) using commercial kits (Randox laboratories Ltd., Llanberis, UK). The levels of serum follicle stimulating hormone (FSH), luteinizing hormone (LH), total testosterone, were measured by respective Centaur CP kits by chemiluminescence immunoassay on an autoanalyzer (ADVIA Centaur CP immunoassay system, Siemens, USA). Commercial kits were used to measure fasting insulin and sex hormone binding globulin (SHBG) levels (Izotop, Budapest, Hungary). In our analyses, the intra-assay and interassay coefficients of variation were 2.8% and 4.5% for total cholesterol, 4.6% and 6.2% for TG, 3.9% and 6.4% for HDL-C, 2.8% and 4.5% for ApoA-1, 3.5% and 4.3% for ApoB, 4.2% and 7.2% for FSH, 3.5% and 6.9% for LH, 8.2% and 8.8% for testosterone, 5.3% and 9.68% for insulin, and 6.1% and 11.89% for SHBG respectively.

Lipid accumulation product (LAP) and visceral adiposity index (VAI) were calculated using formulae as previously described [16,17]. The homeostasis model assessment of insulin resistance (HOMA-IR) and quantitative insulin sensitivity check index (QUICKI) for assessing insulin resistance and sensitivity respectively were determined as earlier [18]. Free and bioavailable testosterone were calculated using total testosterone and SHBG values using a web-based calculator (http://www.issam.ch/freetesto.htm) and FAI was assessed using formula as earlier [18].

### Determination of metabolic syndrome

According to modified American Heart Association/National Heart, Lung, and Blood Institute (AHA/NHLBI) metabolic syndrome is diagnosed by presence of at least three of the following five features: waist circumference ≥ 80 cm, high density lipoprotein (HDL) of <50 mg/dl, triglycerides of ≥150 mg/dl, fasting blood sugar of ≥100 mg/dl, and blood pressure of ≥130/85 mmHg [19].

### Statistical analysis

Prevalence of individual PCOS phenotypes was estimated as percentages. We compared the means of all variables across the groups using Welch one-way analysis of variance (ANOVA) followed by Games-Howell post-hoc test to determine significant pairwise differences between groups. Univariate analysis for comparing the means of variables in lean and obese women of same phenotype was carried out using Independent Samples t-test. All statistical analysis was performed using SPSS v.27 software and P<0.05 was considered statistically significant.

## Results

### Comparison of PCOS related traits amongst different PCOS phenotypes and controls

Clinical, biochemical and hormonal parameters have been compared between different PCOS phenotypic groups, classified according to Rotterdam criteria, and controls (Table 1). The normoandrogenic phenotype D (49.1%), was most prevalent, followed by phenotype A (41.3%), C (5.3%) and B (4.3%). Acanthosis nigricans, a clinical sign of insulin resistance was observed in 54%, 57.1%, 30.8% and 47.1% of women in phenotypes A, B, C and D respectively.

**Table 1:** Clinical, biochemical and hormonal parameters of polycystic ovary syndrome (PCOS) women according to different polycystic ovary syndrome phenotypes and controls.

Variable	A (HA+OA+PCO) (N = 202)	B (HA+OA) (N = 21)	C (HA+PCO) (N = 26)	D (OA+PCO) (N = 240)	Controls (N = 270)
**Age (years)**	25.30±4.81	22.62±3.87 ^a^^,^^i^	26.08±5.47	25.21±4.74	25.66±5.59
**BMI (Kg/m**^**2**^**)**	26.77±5.15	27.06±6.08	25.84±4.47	24±4.23^c^	22.16±4.08^h^^,^^i^^,^^j^^,^^k^
**WHR**	0.82±0.06	0.86±0.08	0.82±0.05	0.81±0.06	0.78±0.06^h^^.^^i^^.^^k^
**FBS (mg/dl)**	89.92±10.98	90.57±15.37	85.31±10.72	88.69±11.11	85.59±8.82^h^^,^^k^
**2h glucose (mg/dl)**	103.56±22.84	110.05±25.62	104.59±17.06	99.27±18.77	91.53±15.25^h^^.^^i^^.^^j^^,^^k^
**Insulin (μIU/ml)**	16.55±8.19	18.73±7.72	14.71±8.27	13.76±7.27^c^	10.64±5.58^h^^.^^i^^,^^k^
**HOMA-IR**	3.72±2.03	4.19±1.90	3.10±1.83	3.01±1.66^c^	2.24±1.21^h^^.^^i^^.^^j^^,^^k^
**QUICKI**	0.32±0.03	0.31±0.02	0.34±0.04	0.33±0.03^c^^,^^e^	0.35±0.03^h^^,^^i^^,^^k^
**FSH (mIU/ml)**	5.84±1.76	5.67±2.17	5.59±2.12	5.79±1.79	6.72±2.31^h^^,^^k^
**LH (mIU/ml)**	10.49±4.99	9.94±6.86	7.99±5.07	8.66±5.12^c^	5.04±2.01^h^^,^^i^^,^^j^^,^^k^
**LH:FSH**	1.93±0.96	1.72±0.81	1.52±0.93	1.58±0.95^c^	0.82±0.39 ^h^^,^^i^^,^^j^^,^^k^
**TT (ng/dl)**	62.03±23.94	56.11±25.13	60.67±22.98	39.38±19.20^c^^,^^e^^,^^f^	37.51±17.51^h^^,^^i^^,^^j^
**SHBG (nmol/l)**	32.06±13.39	30.45±13.85	31.33±15.75	61.74±29.77^c^^,^^e^^,^^f^	84.61±42.60^h^^,^^i^^,^^j^^,^^k^
**Free-T (pmol/l)**	40.36±13.77	37.34±17.14	39.62±11.23	16.85±6.66^c^^,^^e^^,^^f^	13.83±7.39^h^^,^^i^^,^^j^^,^^k^
**Bio-T (nmol/l)**	0.94±0.33	0.88±0.40	0.93±0.26	0.39±0.14^c^^,^^e^^,^^f^	0.33±0.18^h^^,^^i^^,^^j^^,^^k^
**FAI**	7.38±3.05	7.04±3.78	7.56±3.02	2.41±0.92^c^^,^^e^^,^^f^	1.88±1.15^h^^,^^i^^,^^j^^,^^k^
**Total Cholesterol (mg/dl)**	165.06±35.56	171.51±44.31	171.59±40.23	153.87±32.72^c^	149.86±27.53^h^
**HDL-C (mg/dl)**	44.33±15.41	41.6±15.82	51.24±13.62	42.64±13.72^f^	48.40±15.49^h^^,^^k^
**TG (mg/dl)**	102.59±44.53	100.2±51.31	102.06±40.7	90.94±41.73^c^	81±28.28^h^^,^^k^
**LDL-C (mg/dl)**	102.69±33.84	111.94±37.82	99.97±32.55	94.94±29.72^c^	85.08±26.47^h^^,^^i^^,^^k^
**LAP**	32.06±21.21	31.74±21.82	28.49±13.98	22.58±14.82^c^	16.39+11.57^h^^,^^i^^,^^j^^,^^k^
**VAI**	2.17±1.41	2.06±1.32	1.72±0.79	1.95±1.27	1.48±0.76^h^^,^^k^
**ApoA1 (mg/dl)**^g^	107.52±34.78	127.49±37.41	124.33±30	109.21±34.5	117.2±38.39
**ApoB (mg/dl)**^g^	69.35±20.2	72.3±23.52	73.3±17.02	66.96±17.57	63.91±15.95
**ApoB:ApoA1**^g^	0.69±0.25	0.59±0.17	0.62±0.21	0.68±0.28	0.60±0.22^h^
**Metabolic syndrome**	44 (21.8)	9 (42.9)	1 (3.8)	33 (13.8)	9 (3.3)

Data are represented as mean ± SD or N (%) Post-hoc comparisons have been performed by Games-Howell test.

^a^P<0.05 when phenotype A compared with phenotype B.

^b^P<0.05 when phenotype A compared with phenotype C.

^c^P<0.05 when phenotype A compared with phenotype D.

^d^P<0.05 when phenotype B compared with phenotype C.

^e^P<0.05 when phenotype B compared with phenotype D.

^f^P<0.05 when phenotype C compared with phenotype D.

^g^P values obtained for 212 controls, 114 women of phenotype A, 11 women of phenotype B, 22 women of phenotype C and 116 women of phenotype D.

^h^P<0.05 when phenotype A compared with controls.

^i^P<0.05 when phenotype B compared with controls.

^j^P<0.05 when phenotype C compared with controls.

^k^P<0.05 when phenotype D compared with controls.

ApoA-1 = apolipoprotein A-1, ApoB = apolipoprotein B, Bio-T = bioavailable testosterone, BMI = body mass index, FAI = free androgen index, FBS = fasting glucose, Free-T = free testosterone, HDL-C = high density lipoprotein cholesterol, HOMA-IR = homeostasis model assessment for insulin resistance, LAP = lipid accumulation product, LDL-C = low density lipoprotein, QUICKI = quantitative insulin sensitivity check index, SHBG = sex hormone binding globulin, TG = triglycerides, TT = total testosterone, VAI = visceral adiposity index, WHR = waist to hip ratio.

Closer inspection of the table provides insights into detailed differences of PCOS related traits between the different phenotypes as well as control women. The women of only phenotype B were significantly younger than women of phenotype A and controls. Fasting and 2h glucose levels were similar among the four groups indicating glucose metabolism remained unchanged across Rotterdam phenotypes. Post-hoc analyses revealed women of phenotype A were significantly more obese, and had markedly greater insulin resistance and dyslipidemia as evidenced by significantly higher fasting insulin levels and HOMA-IR, significantly elevated LAP, total cholesterol, triglyceride LDL-C levels and ApoB:ApoA-1 ratios, and decreased QUICKI as compared to phenotype D. Additionally, women of Phenotype B also had significantly lower QUICKI versus phenotype D. Interestingly, phenotype C women had highest HDL-C levels amongst all four phenotypes, but this finding was significant only when compared to phenotype D. Phenotype A women also showed significantly increased LH levels and LH:FSH ratios compared to phenotype D women.

As opposed to controls, all phenotypes showed significantly increased BMI, 2h glucose levels, LH levels and LH:FSH ratios, free and bioavailable testosterone levels FAI and LAP and reduced SHBG levels. Only women of phenotypes A and D showed significantly increased fasting glucose, triglycerides and VAI levels, as well as lower HDL-C and FSH levels versus controls. On the other hand, women of A, B, D phenotypes had significantly increased WHR, insulin, HOMA-IR and LDL-C levels and/decreased QUICKI compared to controls. We also noted significant difference in total cholesterol and ApoB:ApoA1 ratio between only phenotype A women and controls.

### Comparison of PCOS related traits in lean and obese women

Obesity is frequently observed in women with PCOS and in our study group, we found that 32.7% and 67.3% of total women with PCOS, and 59.6% and 40.4% of control women were lean and obese respectively. Thus, we observed nearly double the number of obese PCOS women compared to lean in our study. As obesity impacts the clinical, biochemical and hormonal characteristics as well as helps to determine treatment plans, we have also investigated the differences in metabolic and endocrine characteristics across PCOS phenotypes in both lean (Table 2) and obese (Table 3) women with PCOS and BMI-matched controls. The normoandrogenic phenotype D (63.1%) was the most common phenotype in our lean population, followed by A (30%), B (3.8%) and C (3.1%). Contrastingly, in the obese group we found that the prevalence of both phenotypes A and D were relatively close at 46.8% and 42.2% respectively followed by phenotypes C (6.4%) and B (4.6%). Acanthosis nigricans was seen in 33.3%, 33.3%, 0% and 33% of lean women, and 60.4%, 66.7%, 38.1% and 57.2% of obese women with A, B, C and D phenotypes respectively, accentuating that obese women of all phenotypes showed higher incidence of acanthosis nigricans compared to their lean counterparts. Our findings reiterate a previous study by Jones et al., which showed lean women primarily had augmented LHCGR expression and obese women overexpressed insulin receptor in adipose tissue corroborating that the pathophysiology of hyperandrogenemia in PCOS is different in lean and obese women and further confirming that obese women are more insulin resistant [8].

**Table 2:** Comparison of clinical, biochemical and hormonal parameters between lean women with polycystic ovary syndrome (PCOS) according to different phenotypes and lean controls.

Variable	A (HA+OA+PCO) (N = 48)	B (HA+OA) (N = 6)	C (HA+PCO) (N = 5)	D (OA+PCO) (N = 101)	Controls (N = 161)
**Age (years)**	23.19±4.43	21.83±3.49	24.6±5.64	23.95±4.37	24.52±4.65
**BMI (Kg/m**^**2**^**)**	20.55±1.75	20.73±2	18.81±2.13	20.09±2.04	19.46±2.12^h^
**WHR**	0.80±0.06	0.81±0.06	0.79±0.04	0.79±0.06	0.77±0.05
**FBS (mg/dl)**	87.63±7.99	85±16.94	83.80±5.54	85.18±12.14	85.22±8.79
**2h glucose (mg/dl)**	99.67±19.03	103.5±17.55	92.8±10.38	94.62±17.25	90.17±14.70^h^
**Insulin (μIU/ml)**	13.54±6.30	20.58±10.39	11.22±4.72	11.88±6.05	9.54±5.01^h^^,^^k^
**HOMA-IR**	2.93±1.45	4.45±2.65	2.30±0.98	2.46±1.20	1.99±1.06^h^^,^^k^
**QUICKI**	0.33±0.02	0.32±0.02	0.35±0.04	0.34±0.03	0.35±0.03^h^^,^^k^
**FSH (mIU/ml)**	5.66±1.73	4.82±2.30	5.63±2.44	5.93±1.90	6.86±2.54^h^^,^^k^
**LH (mIU/ml)**	12.07±5.87	11.01±8.76	9.42±3.12	9.55±5.22	5.44±2.08^h^^,^^k^
**LH:FSH**	2.23±0.99	2.05±0.91	1.79±0.62	1.70±0.93^c^	0.88±0.41^h^^,^^k^
**TT (ng/dl)**	67.19±26.96	60.78±8.85	69.56±21.63	41.99±20.16^c^^,^^e^	37.46±17.98 ^h^^,^^i^
**SHBG (nmol/l)**	36.56±14.92	37.33±6.17	40.44±16.93	67.53±28.30^c^^,^^e^	86.98±41.03^h^^,^^i^^,^^j^^,^^k^
**Free-T (pmol/l)**	40.45±14.05	35.43±3.14	39.18±12.27	16.40±5.85^c^^,^^e^	13.47±7.49^h^^,^^i^^,^^j^^,^^k^
**Bio-T (nmol/l)**	0.95±0.33	0.83±0.07	0.92±0.29	0.39±0.14^c^^,^^e^	0.32±0.18^h^^,^^i^^,^^j^^,^^k^
**FAI**	6.92±2.73	5.70±0.60	6.41±2.30	2.30±0.87^c^^,^^e^	1.80±1.15^h^^,^^i^^,^^j^^,^^k^
**Total Cholesterol (mg/dl)**	157.79±32.51	184.32±48.78	171.34±14.92	149.28±32.38	148.06±27.55
**HDL-C (mg/dl)**	50.18±14.31	60.20±15.70	57.40±17.96	46.29±12.31	49.21±15.88
**TG (mg/dl)**	86.96±42.20	88.93±55.28	107.10±54.07	79.12+37.34	75.57±25.48
**LDL-C (mg/dl)**	91.16±33.05	108.46±43.45	92.52±8.05	88.33±28.78	83.41±26.34
**LAP**	14.34±10.07	15.23±11.19	13.17±11.41	13.34±8.23	11.43±7.94
**VAI**	1.60±1.24	1.12±0.43	1.66±1.04	1.50±0.85	1.36±0.65
**ApoA1 (mg/dl)**^g^	109.24±37.26	144.24±54.11	118.60±29.23	116.53±33.93	123.05±41.89
**ApoB (mg/dl)**^g^	65.56±18.27	82.84±24.95	75.28±5.61	67.01±16.65	63.63±17.31
**ApoB:ApoA1**^g^	0.65±0.25	0.60±0.16	0.67±0.18	0.63±0.27	0.57±0.22
**Metabolic syndrome**	4 (8.3)	0 (0)	0 (0)	2 (2)	3 (1.9)

Data are represented as mean ± SD or N (%).

^a^P<0.05 when phenotype A compared with phenotype B.

^b^P<0.05 when phenotype A compared with phenotype C.

^c^P<0.05 when phenotype A compared with phenotype D.

^d^P<0.05 when phenotype B compared with phenotype C.

^e^P<0.05 when phenotype B compared with phenotype D.

^f^P<0.05 when phenotype C compared with phenotype D.

^g^P values obtained for 35 women of phenotype A, 5 women of phenotype B, 5 women of phenotype C 64 women of phenotype D and 124 controls.

^h^P<0.05 when phenotype A compared with controls.

^i^P<0.05 when phenotype B compared with controls.

^j^P<0.05 when phenotype C compared with controls.

^k^P<0.05 when phenotype D compared with controls.

ApoA-1 = apolipoprotein A-1, ApoB = apolipoprotein B, Bio-T = bioavailable testosterone, BMI = body mass index, FAI = free androgen index, FBS = fasting glucose, Free-T = free testosterone, HDL-C = high density lipoprotein cholesterol, HOMA-IR = homeostasis model assessment for insulin resistance, LAP = lipid accumulation product, LDL-C = low density lipoprotein, QUICKI = quantitative insulin sensitivity check index, SHBG = sex hormone binding globulin, TG = triglycerides, TT = total testosterone, VAI = visceral adiposity index, WHR = waist to hip ratio.

**Table 3:** Comparison of clinical, biochemical and hormonal parameters between obese women with polycystic ovary syndrome (PCOS) according to different phenotypes and obese controls.

Variable	A (HA+OA+PCO) (N = 154)	B (HA+OA) (N = 15)	C (HA+PCO) (N = 21)	D (OA+PCO) (N = 139)	Controls (N = 109)
**Age (years)**	25.96±4.75	22.93±4.08	26.43±5.51	26.13±4.81	27.36±6.42^i^
**BMI (Kg/m**^**2**^**)**	28.71±4.23	29.59±5.22	27.51±2.97	26.82±2.96^c^	26.15±2.79^h^
**WHR**	0.83±0.06	0.87±0.04^e^	083±0.05	0.82±0.05	0.80±0.05^h^^,^^i^
**FBS (mg/dl)**	90.64±11.69	92.80±14.71	85.67±11.69	91.24±9.55	86.15±8.87^h^^,^^k^
**2h glucose (mg/dl)**	104.79±23.85	112.67±28.32	107.54±17.29	102.71±19.16	93.53±15.9^h^^,^^j^^,^^k^
**Insulin (μIU/ml)**	17.49±8.49	17.99±6.67	15.54±8.79	15.13±7.79	12.27±5.98^h^^,^^i^^,^^k^
**HOMA-IR**	3.97±2.12	4.09±1.61	3.30±1.95	3.41±1.82	2.62±1.34^h^^,^^i^^,^^k^
**QUICKI**	0.32±0.03	0.31±0.02	0.33±0.04	0.33±0.03	0.34±0.03^h^^,^^i^^,^^k^
**FSH (mIU/ml)**	5.90±1.77	6.01±2.09	5.57±2.10	5.69±1.70	6.51±1.91^k^
**LH (mIU/ml)**	10 ±4.6	9.51±6.25	7.66±5.43	8.01±4.97^c^	4.46±1.76^h^^,^^i^^,^^k^
**LH:FSH**	1.84±0.93	1.58±0.76	1.46±0.99	1.49±0.95^c^	0.73±0.34^h^^,^^i^^,^^j^^,^^k^
**TT (ng/dl)**	60.42±22.78	54.24±29.35	58.55±23.28	37.49±18.31^c^^,^^f^	37.58±16.88^h^^,^^j^
**SHBG (nmol/l)**	30.66±12.60	27.69±15.23	29.17±15.07	57.54±30.20^c^^,^^e^^,^^f^	81.11±44.79^h^^,^^i^^,^^j^^,^^k^
**Free-T (pmol/l)**	40.34±13.73	38.10±20.35	39.72±11.29	17.18±7.19^c^^,^^e^^,^^f^	14.36±7.24^h^^,^^i^^,^^j^^,^^k^
**Bio-T (nmol/l)**	0.94±0.33	0.89±0.48	0.93±0.26	0.39±0.15^c^^,^^e^^,^^f^	0.34±0.17^h^^,^^i^^,^^j^^,^^k^
**FAI**	7.53±3.14	7.57±4.38	7.85±3.15	2.49±0.95^c^^,^^e^^,^^f^	1.99±1.15^h^^,^^i^^,^^j^^,^^k^
**Total Cholesterol (mg/dl)**	167.32±36.26	166.39±43.09	171.65±44.48	157.20±32.68	152.52 ±27.40^h^
**HDL-C (mg/dl)**	42.50±15.33	34.16±7.87^a^^,^^d^	49.77±12.48 ^f^	39.99±14.12	47.20±14.90^i^^,^^k^
**TG (mg/dl)**	107.46±44.24	104.70±50.92	100.86±38.45	99.53±42.77	89.02±30.36^h^
**LDL-C (mg/dl)**	106.29±33.39	113.33±36.90	101.74±35.98	98.01±29.83	87.53±26.60^h^^,^^k^
**LAP**	37.58±20.75^c^	38.35±21.72	32.14±12.06	29.29±14.95	23.71±12.20^h^^,^^j^^,^^k^
**VAI**	2.35±1.42^b^	2.43±1.37	1.73±0.75	2.28±1.41	1.66±0.87^h^^,^^k^
**ApoA1 (mg/dl)**^g^	106.75±33.83	113.53±15.8	126.01±30.9^f^	100.21±33.34	108.97±31.25
**ApoB (mg/dl)**^g^	71.03±20.90	63.52±20.12	72.72±19.25	66.90±18.83	64.30±13.87
**ApoB:ApoA1**^g^	0.71±0.24	0.58±0.20	0.61±0.23	0.74±0.28	0.64±0.21
**Metabolic syndrome**	40 (26)	9 (60)	1 (4.8)	31 (22.3)	6 (5.5)

Data are represented as mean ± SD or N (%).

^a^P<0.05 when phenotype A compared with phenotype B.

^b^P<0.05 when phenotype A compared with phenotype C.

^c^P<0.05 when phenotype A compared with phenotype D.

^d^P<0.05 when phenotype B compared with phenotype C.

^e^P<0.05 when phenotype B compared with phenotype D.

^f^P<0.05 when phenotype C compared with phenotype D.

^g^P values obtained for 79 women of phenotype A, 6 women of phenotype B, 17 women of phenotype C, 52 women of phenotype D and 88 controls.

^h^P<0.05 when phenotype A compared with controls.

^i^P<0.05 when phenotype B compared with controls.

^j^P<0.05 when phenotype C compared with controls.

^k^P<0.05 when phenotype D compared with controls.

ApoA-1 = apolipoprotein A-1, ApoB = apolipoprotein B, Bio-T = bioavailable testosterone, BMI = body mass index, FAI = free androgen index, FBS = fasting glucose, Free-T = free testosterone, HDL-C = high density lipoprotein cholesterol, HOMA-IR = homeostasis model assessment for insulin resistance, LAP = lipid accumulation product, LDL-C = low density lipoprotein, QUICKI = quantitative insulin sensitivity check index, SHBG = sex hormone binding globulin, TG = triglycerides, TT = total testosterone, VAI = visceral adiposity index, WHR = waist to hip ratio.

Specifically, lean phenotype D women had significantly lower LH:FSH ratios versus lean phenotype A, and total, free and bioavailable testosterone concomitant with higher SHBG levels compared to lean women of phenotype A and B. We had very low sample numbers in lean phenotypes B and C which may explain why significance was not seen in post-hoc analyses despite phenotype B having highest insulin levels, HOMA-IR and lowest QUICKI, or phenotype C also having increased free, bioavailable and total testosterone and FAI compared to phenotype D.

Alternatively, in the obese group, women with phenotype D had significantly lower WHR compared to phenotype B. Moreover, obese women of phenotype D presented with distinctly decreased circulating LH levels and LH:FSH ratio compared to phenotype A. Further, total testosterone levels were significantly lower in phenotype D women as opposed to phenotypes A and C only, while all other androgen parameters showed significant differences with A, B and C phenotypes. Amongst lipid related traits, we report that phenotype B showed significantly reduced HDL-C levels compared to phenotypes A and C, while phenotype C women had significantly elevated HDL-C and ApoA-1 levels compared to phenotype D women. Furthermore, phenotype A women in obese group showed significantly increased LAP and VAI when compared to phenotypes D and C respectively.

Simultaneously, when lean PCOS phenotypes were compared to lean controls, significantly aggravated glucose, insulin, gonadotropin traits could be seen in lean A and D phenotypes. Amplified androgen traits excepting total testosterone were observed in all 4 phenotypes, compared to lean controls, which showed significant difference only with phenotypes A and B. If we now turn to the obese group, phenotype B women were younger and had higher WHR than controls. Fasting glucose levels were lower in obese controls only compared to A and D phenotypes while 2 h glucose levels were decreased when compared to A, C and D phenotype women. Interestingly, significant differences for insulin related traits were observed when comparing obese controls with obese women of A, B and D phenotypes. While controls are significantly higher FSH levels versus D phenotype, LH levels were only significantly increased when compared to A, B, D phenotypes. Moreover, all four phenotypes showed markedly increased LH:FSH ratios, free and bioavailable testosterone levels and FAI with reduced SHBG levels when compared to controls. Moreover, worsened dyslipidemia was seen in all PCOS phenotypes in obese group versus obese controls. Particularly significantly increased total cholesterol, triglycerides, LDL-C levels, LAP and VAI were observed in phenotype A as opposed to controls. Phenotype D also showed dyslipidemia compared to controls marked by significantly higher LAP, VAI and LDL-C levels with decreased HDL-C levels. Phenotype B women and phenotype C also had lower HDL-C and increased LAP respectively compared to obese controls.

In summary these results indicate that few PCOS related traits show differences across phenotypes when BMI matched. Importantly, we also note that both obesity and PCOS status impact phenotypic manifestation as substantiated by our findings that obese PCOS phenotypes show worsened cardiometabolic patterns compared to obese controls.

### Comparison of PCOS related traits between individual PCOS phenotypes according to obesity

Further, we have compared traits between lean and obese women for each individual PCOS phenotype (Table 4) which reveals that intriguingly cardiometabolic traits are exacerbated in obese women even when matched for phenotype. This trend was most prominently observed in phenotypes A and D.

**Table 4:** Comparison of lean vs. obese women in each individual phenotype (p-values only).

Variable	Lean A vs. Obese A	Lean B vs. Obese B	Lean C vs. Obese C	Lean D vs. Obese D
**WHR**	**0.002**	0.576	0.118	**<0.0001**
**FBS (mg/dl)**	**0.046**	0.305	0.734	**0.001**
**2h glucose (mg/dl)**	0.176	0.473	0.084	**0.001**
**Insulin (μIU/ml)**	**0.001**	0.501	0.303	**<0.0001**
**HOMA-IR**	**<0.0001**	0.703	0.283	**<0.0001**
**QUICKI**	**0.011**	0.728	0.476	**<0.0001**
**FSH (mIU/ml)**	0.427	0.265	0.955	0.364
**LH (mIU/ml)**	**0.029**	0.663	0.496	**0.021**
**LH:FSH**	**0.013**	0.240	0.489	0.073
**TT (ng/dl)**	0.087	0.446	0.346	0.064
**SHBG (nmol/l)**	**0.007**	0.154	0.154	**0.010**
**Free-T (pmol/l)**	0.959	0.629	0.925	0.416
**Bio-T (nmol/l)**	0.840	0.618	0.935	0.676
**FAI**	0.230	0.126	0.912	0.121
**Total Cholesterol (mg/dl)**	0.105	0.454	0.988	0.055
**HDL-C (mg/dl)**	**0.002**	**<0.0001**	0.269	**<0.0001**
**TG (mg/dl)**	**0.005**	0.538	0.765	**<0.0001**
**LDL-C (mg/dl)**	**0.007**	0.798	0.580	**0.010**
**LAP**	**<0.0001**	**0.024**	**0.004**	**<0.0001**
**VAI**	**0.001**	**0.004**	0.856	**<0.0001**
**ApoA1 (mg/dl)**	0.727	0.214	0.639	**0.011**
**ApoB (mg/dl)**	0.184	0.188	0.776	0.972
**ApoB:ApoA1**	0.252	0.826	0.607	**0.032**

Note: Significant differences (P<0.05) are indicated in bold.

ApoA-1 = apolipoprotein A-1, ApoB = apolipoprotein B, Bio-T = bioavailable testosterone, FAI = free androgen index, FBS = fasting glucose, Free-T = free testosterone, HDL-C = high density lipoprotein cholesterol, HOMA-IR = homeostasis model assessment for insulin resistance, LAP = lipid accumulation product, LDL-C = low density lipoprotein, QUICKI = quantitative insulin sensitivity check index, SHBG = sex hormone binding globulin, TG = triglycerides, TT = total testosterone, VAI = visceral adiposity index, WHR = waist to hip ratio.

#### Prevalence of metabolic syndrome and its components in PCOS phenotypes

**Fig 1:**
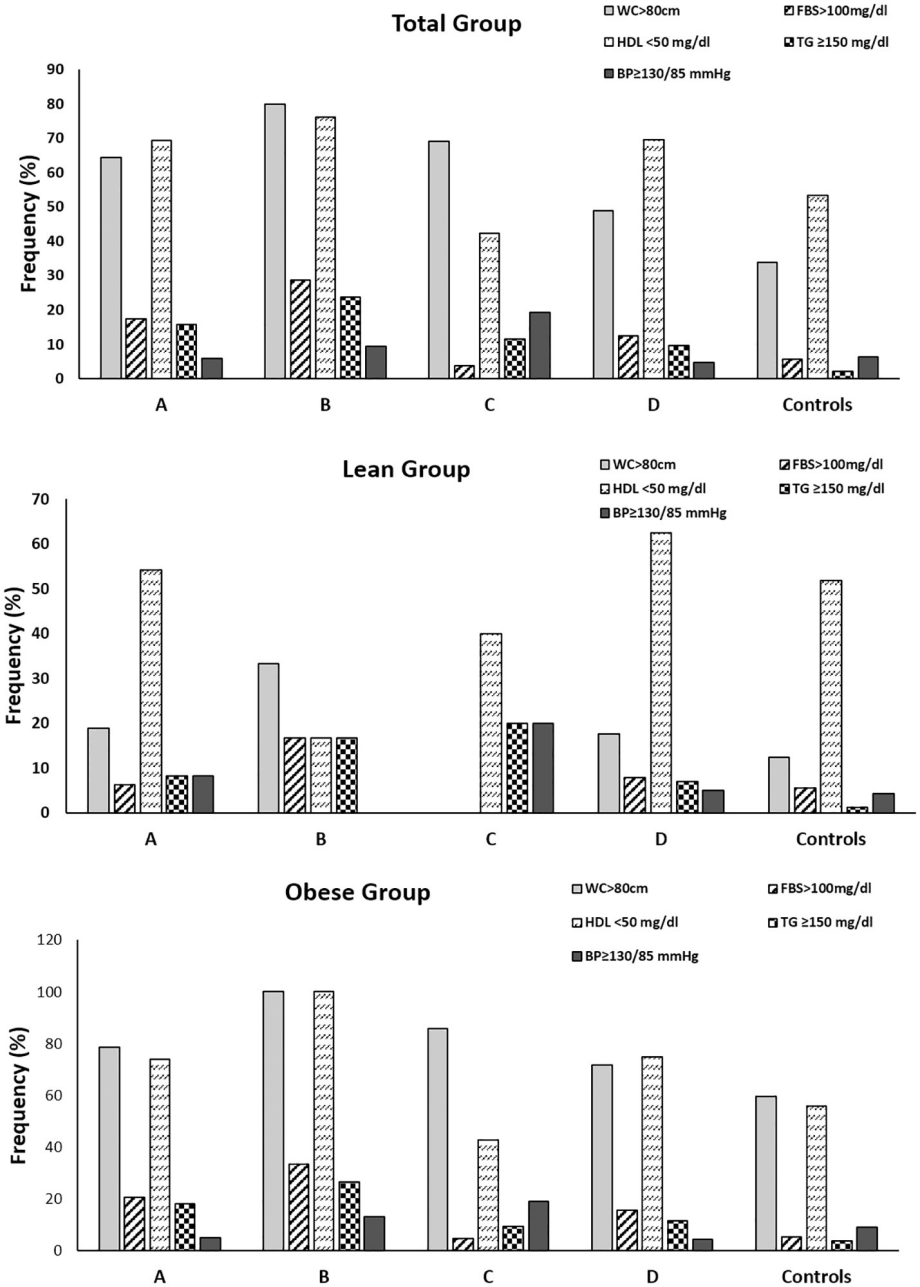
Prevalence of components of metabolic syndrome in women with PCOS and controls. Frequency of the components of metabolic syndrome in total (A), lean (B) and obese (C) women with PCOS and controls. BP = blood pressure; FBS = fasting blood sugar; HDL = high density lipoprotein; MS = metabolic syndrome; TG = triglycerides; WC = waist circumference.

We noted that, overall, metabolic syndrome prevalence was higher in PCOS women (17.8%) compared to controls (3.3%). Also, we noted that metabolic syndrome was present in 42.9% of women of phenotype B, 21.8% of women of phenotype A, 13.8% of women of phenotype D and 3.8% of phenotype C. (Table 1). In lean women, only 8.3% of phenotype A, and 2% of phenotype D showed metabolic syndrome but none in phenotype B or C (Table 2). Conversely, obese women of phenotype B (60%) had highest frequency of metabolic syndrome followed by A (26%), D (22.3%) and C (4.8%) respectively (Table 3). Moreover, we found that reduced HDL-C and elevated waist circumference were most prevalent metabolic syndrome components across all phenotypes regardless of metabolic syndrome presence or obesity status (Fig 1).

## Discussion

In the current study, we have observed that there is greater predisposition towards normoandrogenic phenotype D characterized by milder metabolic and androgen profile in our study population from Western India. Furthermore, classification into lean and obese groups showed that cardiometabolic profiles are similar when PCOS phenotypes were BMI-matched, but all PCOS women present with significant metabolic and endocrine dysfunction compared to BMI-matched controls.

The inclusion of polycystic ovaries into NIH criteria provides the basis for sub-categorizing patients into four main groups and a recent NIH panel has recommended these criteria for clinicians [9,20]. While phenotypes A, B and C are classified as hyperandrogenic phenotypes, phenotype D represents anovulatory non-hyperandrogenic subset, underscoring that PCOS is also a metabolic disorder [9]. Thus, it is imperative to delineate the phenotypic heterogeneity often observed in PCOS to suitably tailor therapy for minimizing the risk of reproductive and cardiometabolic complications in future. In our study population we found that phenotype D is most prevalent, similar to Vietnamese [21] and Chinese [22] population. In contrast, phenotype A was predominant in Greek [23,24], women from Boston and Iceland [25], Canadian [26], Italian [27], French [28], British [29], Polish [30], Croatian [31], Chinese [32], Iranian [33], Korean [34,35], Turkish [36,37] and Bulgarian [38] PCOS populations. Interestingly, other studies have reported increased prevalence of phenotype B in Iranian [39], and phenotype C in Iranian [40] and Chinese [41] PCOS women. The phenotype and genetic makeup of Indian women with PCOS will expectedly be different from Asian, African and European populations. What is most intriguing is that studies from India itself demonstrate diverse results as seen by prevalence of different phenotypes amongst different ethnic populations across India. Previous studies from Mumbai in adolescent population and North India also report maximum prevalence of phenotype D in line with our present study, while yet another study from North India stated phenotype C to be most common [42–44]. In contrast, other groups again from North India [45–47] as well as East India [48,49] have detailed increased frequency of phenotype A in their populations. Together, the abovementioned studies emphasize the impact of ethnic, geographic and genetic variation on phenotypic presentations of PCOS.

In our study, we noted that phenotype D women represented a milder cardiometabolic profile in terms of abdominal adiposity, pro-atherogenic dyslipidemia, aggravated insulin resistance, abnormal gonadotropins, and hyperandrogenism compared to other PCOS phenotypes as corroborated in previous studies [22,30,48,50,51]. Similar findings were reported in Iranian [40], Bulgarian [38], Italian [27], Canadian [26], North Indian [45], East Indian [49], Chinese [22], Korean [34,35], and Turkish [36] women with PCOS. Another recent study in Mediterranean women with PCOS reported most adverse metabolic pattern in phenotype B followed by phenotype A, moderate glucose dysmetabolism and dyslipidemia in phenotype C and no metabolic abnormalities in phenotype D [52]. Surprisingly, in South-west Chinese women, phenotype D was reported to be associated with highest prevalence of dyslipidemia and metabolic syndrome [53]. In sharp contrast, no differences in clinical, metabolic or endocrine characteristics among four different phenotypes were reported in Chinese [32] and Iranian [39] women.

Obesity impacts both reproductive and metabolic anomalies associated with PCOS [3]. This is why we believe it is imperative to delineate the influence of obesity status on the severity of metabolic and hormonal manifestations in these Rotterdam phenotypes which may help to minimize cardiometabolic and reproductive long-term consequences. Towards this, we have classified our study group into lean and obese women and compared PCOS related traits amongst different PCOS phenotypes and BMI-matched controls. Lean women predominantly presented with D phenotype, while frequency of A and D phenotypes were almost comparable in obese women with PCOS. Both lean and obese women with phenotype D show adverse gonadotropin and cardiometabolic profiles compared to BMI-matched controls indicating that normoandrogenic phenotype D women form an important part of the PCOS spectrum and also necessitate monitoring of endocrine and cardiometabolic risk factors to impede harmful cardiovascular and reproductive outcomes. Intriguingly, dysglycemia was not disparate amongst PCOS phenotypes in either lean or obese subgroups indicating that derangements in glucose metabolism are intrinsic to PCOS pathogenesis. Our findings are similar to an earlier study in Greek women which demonstrated that there were no differences in markers of insulin resistance between phenotypes A with B and C, and C with B and D [24] in the lean group. On the other hand, they reported that lean women with phenotype A and B had greater AUC gluc-OGTT and lower glucose/insulin compared to women with phenotype D [24]. There is a growing body of literature that recognizes the impact of two major surrogate markers, LAP and VAI for determining fat distribution. A number of studies have reported increased values in women with PCOS [54,55] and have also suggested an association of these indices with impaired glucose tolerance [56], metabolic syndrome [57–59], and anovulation [4,60]. Another important finding of our study was LAP and VAI were comparable amongst PCOS phenotypes in the total and lean groups, but in the obese group phenotype A showed markedly higher LAP and VAI compared to phenotypes D and C respectively. In addition, recent studies from India have shown that the classic hyperandrogenic phenotypes of PCOS present with elevated VAI [42,61]. Lack of more significant differences in metabolic traits across PCOS phenotypes in obese group suggests that obesity may mask differences in intrinsic harmful metabolic profiles. Interestingly, obese phenotype C women presented with maximum HDL-C levels compared to other phenotypes, indicating phenotype C may have more favourable cardiovascular profile along similar lines to previous studies which have shown that ovulatory PCOS women had milder dyslipidemia with decreased risk of CVD [62,63]. However, this did not show significance in post-hoc analyses. Contrarily phenotype B was associated with the lowest HDL-C levels indicating that the classic PCOS group was most prone to dyslipidemia. On the other hand, comparison between obese Greek women showed that women with phenotype A had lower QUICKI than phenotype B while women of both phenotype A and D had higher insulin levels and HOMA-IR with lower glucose/insulin and QUICKI compared to phenotype C, while insulin resistance was comparable between phenotypes A and D, B and C, and B and D [24].

In addition, comparison of endocrine and metabolic factors between lean and obese women belonging to the same Rotterdam phenotype reveals that majority of insulin, gonadotropin and lipid related factors are worsened by obesity which was largely discerned in phenotypes A and D. All in all, it may seem that obesity may not shape androgen profiles as no significant differences were noted in total, free and bioavailable testosterone levels between lean and obese women belonging to the same phenotype. Interestingly, only obese women of phenotype D show aggravated glucose intolerance as indicated by poorer 2 h glucose levels following OGTT, and significantly reduced ApoA-1 levels coupled with increased ApoB:ApoA1 ratios compared to lean counterparts. Therefore, despite belonging to the same phenotype, obesity contributes to more unfavourable cardiometabolic outcomes, and this effect is more severe in normoandrogenic phenotype D women which is considered to be towards the milder end of the PCOS spectrum.

Metabolic syndrome represents a set of cardiometabolic risk factors, several of which are shared with PCOS, which intensifies their susceptibility to adverse cardiovascular events [64]. Intriguingly, other studies in Indian population reported markedly greater prevalence rates ranging between 30–53% [43,45,65–69] in contrast to our finding of approximately 18% frequency in PCOS women overall. Lifestyle and dietary factors, genetic influences, ethnic and geographic diversity and obesity status may contribute to the variations observed in the incidence of metabolic syndrome. We found that the Rotterdam classification also influences the incidence of metabolic syndrome in PCOS women with maximum prevalence being observed in women of phenotype B, followed by phenotypes A, D and C. Similarly, highest risk of metabolic syndrome was revealed in phenotypes A and B in Turkish [59,70], Indian [43,45,48,49], Iranian [40], Chinese [22], Greek [71], Brazilian [72], Dutch [73] women with PCOS. Thus, hyperandrogenic phenotypes present with worsened metabolic parameters and have higher risk of metabolic syndrome development. However, we noted that no metabolic syndrome was reported in phenotypes B and C with phenotype A having highest frequency in lean women. In contrast amongst obese women, phenotypes A and D have nearly comparable incidence of metabolic syndrome. This accentuates that obesity also impacts prevalence of metabolic syndrome in these phenotypes.

The relationship of PCOS and obesity is highly complex and obesity is known to exacerbate risk of impaired glucose tolerance, dyslipidemia, metabolic syndrome and hyperandrogenemia by concomitantly suppressing hepatic SHBG synthesis. Our study is strengthened by comparing PCOS phenotypes with BMI matched controls to highlight significance of obesity in influencing PCOS related traits along with phenotype classification. In essence, our study emphasizes that obesity may mask differences in metabolic parameters across phenotypes. Our findings also reiterate that cardiometabolic dysfunction is heavily influenced by obesity as despite being classified into same Rotterdam phenotype, obese women still show significant insulin resistance and dyslipidemia for phenotypes A and D. As obesity is seen to play a considerable role in development of metabolic changes in affected women, simply classifying according to Rotterdam criteria is not sufficient for predicting long term outcomes in women with PCOS. Moreover, we observed that low HDL-C and increased waist circumference were the most frequent amongst individual metabolic syndrome components across all phenotypes and regardless of BMI classification, suggesting that these women are vulnerable to insulin resistance and cardiovascular complications.

A major limitation of our study was the inability to estimate exact Ferriman-Gallwey scores as patients were cosmetically conscious and utilized remedies for correction of the same. Thus, we could only record yes/no answers based on patient responses. It has been demonstrated that self-reporting based on patient perception of clinical signs of hyperandrogenism has proven to be unreliable, which is why we have utilized the 95^th^ percentile of FAI as a cutoff for hyperandrogenism as diagnostic criterion to strengthen the diagnostic efficiency of our study. Moreover, we have not found it feasible to perform liquid chromatography with tandem mass spectrometry measurements for testosterone levels in the present study which is highly discerning and sensitive, at even low levels. Another important limitation is that although we have utilized Welch F test and Games Howell post-hoc tests to minimize error, results for groups B and C must still be prudently examined and interpreted with caution owing to low numbers in these groups, particularly for lean phenotype B and C group.

Collectively, our study findings reveal the importance of categorizing all women with PCOS into different phenotypic groups to recognize the milder forms of PCOS which would otherwise be missed using more stringent diagnostic criteria. Additionally, obesity is an important determinant of PCOS related traits and BMI-based classification may further improve our understanding of pathogenic pathways, long-term risks and guide treatment options and monitor outcomes.

## Conclusion

The present study offers the opportunity to advance the understanding of complexity of PCOS pathogenesis by demonstrating the distinct hormonal and metabolic makeup of each subtype with phenotype A presenting the most adverse cardiometabolic profile amongst all PCOS phenotypes. We observed that phenotype D is the most prevalent subtype in our study population from Western India, which would have otherwise been missed in absence of using Rotterdam criteria for diagnosis of PCOS. Although milder amongst PCOS phenotypes, our study highlights that they still present with adverse clinical patterns compared to controls, indicating that this phenotype also demands timely monitoring and therapeutic intervention to impede unfavourable reproductive and cardiometabolic sequalae. Furthermore, we show that obesity masks differences in intrinsic harmful metabolic profiles across PCOS phenotypes. Lack of substantial variation in cardiometabolic traits across phenotypes when classified according to BMI, highlights that presence of obesity is a key influencer, and should be considered in tandem with Rotterdam classification for understanding PCOS perturbations in order to tailor effective management therapy. Future work is warranted to establish phenotypic classification in ethnically diverse populations in India to build a comprehensive clinical profile on the national level that will aid in formulation and implementation of appropriate treatment strategies unique to each woman’s phenotype.
